# Mr. Dunning, on the Vaccine Innoculation

**Published:** 1800-05

**Authors:** Richard Dunning


To Drs. JENNER, PEARSON, and WOODVILLE.

GENTLEMEN,

THE Small-Pox, which, previously to the introduction of inoculation, appeared only at intervals, and, from the great care to avoid them, were often local, are now become so frequent and general, it is, I believe, an acknowledged fact, that a greater number of persons are destroyed by them in a given time than before that remarkable discovery. The great DESIDERATUM of their extinction exists, therefore, at this time at least, in all its force. Some ingenious men have not only thought the attainment of this great event possible, but have absolutely thrown out some hints, and even recommended certain regulations for that purpose, which, if they had been fully acted on, would perhaps have gone near to be succesful: surely, then, with the agency of Cow-pox at our command, which seems to offer an obvious, a ready, and practicable means, and before which all human arrangements sink into futility—the pursuit is neither visionary nor romantic. This is not a wonder-working attempt to alter the structure and constitution of the human frame, or change the nature of the elements which surround us, but merely an endeavour to destroy the locality of a specific infection, or rather to intercept its action, by interposing another, of a character infinitely more benign; another, which affords complete security against every future exposure to the other; in general, produces little more than a slight ephemeral indisposition, and rather corrects, there is reason to believe, than leaves behind it, any ill effects; which is only communicable through the medium of inoculation, and of course will cease, in a great measure, to be a human disease the moment the inoculation of it ceases to be necessary; and this is a consequence, I confess, I am sanguine enough to anticipate. To be completely succesful, we want only, I think, weapons of equal efficacy wherewith to controul popular and imaginary prejudice, and silence unfounded and ill-natured objections.

The human mind, happily for the world, is so constituted, it always meets at first new propositions, and even new truths, with great caution and reluctance; and, on this account, it has for the most part been found indispensibly necessary, frequently and familiarly to present them, in order that they may be fully admitted and received: this however, at last, with but few exceptions, usually happens. As general and repeated exertion will then do much, it certainly behoves medical men to co-operate co-operate in the profecution of a queftion, indifputably the moft important in the hiftory of medicine. I have ventured to reflect on this fubject; and the refult has been, to fuggeft to you a belief, indeed a conviction, (I am authorized to fay that Doctors Remmett and Woolcomb, two very eminent and refpectable phyficians here; Dr. Huggan, Weft Kent; and many other medical friends, think fo too) that if the annexed queries, or fomething like them, were fo anfwered in this Journal, as that Extracts from hence might be inferted in fome more general vehicle of intelligence than a medical publication, it would be one, perhaps *the moft effectual*, preliminary means, of placing this interefting object, as it were, in a focus, under the moft ftriking point of view, and of forcing and fixing it on public attention; and for this reafon, beft calculated to leffen apprehenfion, and counteract unjuft and indifcriminate oppofition. I believe the greateft part of thefe queftions have, indeed, been fully adverted to, and very fatisfactorily anfwered in feveral valuable publications, for which the medical world is indebted to your refpective exertions. But medical books, generally fpeaking, fall into medical hands only; and it is as much impoffible for practitioners to carry about and read them to their friends and patients, as it would be tedious and unpleafant for thefe to hear. Befides, certain it is, that whatever falls under the public eye, through the medium of the public papers, arrefts more forcibly public obfervation, and longer detains it. To an inquiry of fuch great and general intereft to fociety, it is not unreafonable, I believe, to expect that the Editors of the public prints will, with great willingnefs, appropriate a column, now and then, in their refpective papers.

Should the abolition of the Small-pox at laft be effected, (and with our prefent profpects, I repeat it, there is neither enthufiafm nor prefumption in the expectation) the many beneficial confequences that would refult from it to mankind are incalculable. One infallible effect of it would be, that the vaft chafm in population, made by the prefent depopulating war, would in a few years be filled up; another, that the greateft fource of domeftic affliction would be removed. Until, then, this dreadful difeafe fhall have difappeared for fome years in every part of Europe, I hope, and really flatter myfelf, it will fhortly be confidered as much a matter of courfe, and be felt as much a duty, to inoculate little folks with the Cow-pox in their earlieft infancy, (provided nothing unfortunate occurs in the mean time in our experience with it) as it is now to give them a name.

Under thefe impreffions, and convinced alfo of the many prefent advantages attending the vaccine, it fhould be called the JENNERIAN Inoculation: I recommended and introduced it, as far as I have been able, into my own practice; than which, as far as it is gone, nothing can have succeeded better. But I have had, and still have, much opposition to contend with here. Among many others which I have had to combat, the following objections (and these are sometimes started too by medical men) are the most powerful, and most frequently urged, viz. That the communication of a bestial disease must always be naturally abhorrent to human feelings;* that it may call

* From the ready admission of cow-pox into the human body, it is no violent supposition, that the small-pox, measles, and some other diseases, are all of bestial origin. We have always believed they did not exist ab origine, because we have not been able to discover the smallest reference to them in the numerous writings of the ancient Physicians and Naturalists, whose descriptions and histories of diseases, unbiassed as they were by the proteus of theory, and whose attentions to natural productions were certainly not less accurate and indefatigable than those of the most correct and industrious of the moderns, and which have therefore secured to them the unequivocal approbation, thus far, of every succeeding age; it seems more reasonable to conclude, at this time, that they were impressions ab externo, than that they should have been indigenous to the human constitution, and from some unknown and inexplicable cause, were not elicited till after a lapse of several thousand years.

Does not this idea receive much countenance from the swine and the chicken pox? From what source were derived those diseases, and their names? Were not these diseases formerly conveyed by some accident or other from the swine and the fowl, and along with them their names? Or were they originally human, but predestined however to remain *morbi innominati*, until a something should appear in the brute creation bearing a striking resemblance to them, whence they might be designated? Risum teneatis amici! Obviously, the terms imply priority of existence of those diseases in the two animals.——Assuming, then, by an easy and just analogy, what perhaps will, bye and bye, be readily enough conceded, viz. that these diseases did draw their origin from brutes: If we reflect for a moment on the very simple characters of these complaints as we now have them, that the treatment of them is so trifling, it becomes in the greater number of instances more immediately the province of the good woman or her nurse, than an object of medical attention, and that no unpleasant concomitants attended, or rather followed their introduction, (certainly, none have ever been attributed to it); if we compare the different habitudes and dispositions of the cow and the hog, the result of such comparison is not likely to be a very generally received opinion, that these of the latter are in many respects more desirable and engaging than those of the former; if we recollect that it has lately been recommended, from great authority, to patients labouring under pulmonary complaints, to become even inmates of the cow; that the flesh of this sleek and wholesome animal makes a large portion of our daily food; that her milk, unquestionably the most bland and congenial in the whole range of human diet, is absolutely formed in and secreted from the immediate and only seat of cow-pox; that from this circumstance it must necessarily have happened, in a very great number of instances, that pretty large portions of its virus have been, sit venia verbo, literally eaten and drank; and that hence call into action conſtitutional affections, may produce others of a new and anomalous character; that theſe may prove unmanageable and of difficult cure; may poſſibly, at laſt, leave behind them indelible bad impreſſions in the habit; and after all this, the conſtitution not ſecured againſt future expoſure to the Small-pox. Theſe, Gentlemen, are ſome of the circumſtances, together with an earneſt deſire to throw in my mite of contribution on this important occaſion, that led to the following queries, which I have taken the liberty to addreſs to you; and, indeed, to whom could I direct them with ſo much propriety as to three gentlemen, the firſt of whom continues with equal induſtry and ſucceſs his ingenious inveſtigations on this great queſtion, which he had the honour to announce to the world; (an event, which will give an illuſtrious immortality to his name, and would alone render memorable the cloſe of the eighteenth century) and of whom the two latter, have on a very large ſcale, laboured in the ſame field of inquiry with the greateſt judgment and ability, and on whoſe deciſions, from their long experience and great medical reputation, the public will be encouraged to rely with almoſt implicit confidence?

If I am fortunate enough to find you coinciding with me in this propoſal, and your extenſive engagements will admit of its early adoption, I am perſuaded it will at once be, in no ſmall degree, uſeful in this large town; for the Small-pox hav-

---

hence it might have been expected, were it in any great degree inimical to the healthy œconomy of the human conſtitution, that its deleterious effects would have been on thoſe occaſions frequently and ſtrikingly exerted — but we hear of no ſuch deleterious conſequences, becauſe none ſuch, I preſume, have ever been obſerved: In ſhort, if we allow ourſelves to review all, or even ſome of theſe circumſtances, certain it is there will be very little danger to be apprehended from the cow-pock virus — an unmerited name — when externally forced on the ſyſtem through the cutaneous abſorbents; and as certain it is that we ſhall hear, very ſhortly, leſs and leſs of its beſtiality, and all the terrible things which attach to it; thoſe terrible things which are ſo often, with an unaccountable induſtry, preſſed into the ſervice of unrelenting prejudice, and, ſeemingly with a ſhow of triumph, brought againſt this gentle, unoffending, unintruſive, and benevolent ſtranger, that aſſuredly will always withdraw itſelf in that moment in the which it is neglected: Oh tempora! oh mores!

The poſſibility of the above-mentioned diſeaſes having had their riſe in a way ſomewhat ſimilar to the inſinuation of the variolæ vaccinæ into the human body, goes far to ſtrengthen the poſſibility, and even probability, that they will again be all ultimately removed from it; and that theſe events will perhaps be attained through the agency of ſome ſuch medium, as that we now propoſe to employ with ſo promiſing a proſpect of ſucceſs, for the annihilation of the worſt of them; at any rate, it is certainly an important ſubject of inveſtigation, and I ſhall be extremely glad to ſee it taken up by ingenious men. —Quere, Does the cow-pox protect from meaſles?

 ing ing hardly made their appearance with us this year, and inoculation for them not yet begun, we ſhould certainly be enabled to anticipate them, at leaſt in ſome inſtances, and of courſe ſave many lives. I have the honour to be,

GENTLEMEN,

With the greateſt reſpect,

Your moſt obedient humble ſervant,

RICHARD DUNNING.

### QUERIES.

1. Is not the inoculated cow-pox, almoſt without an exception, a diſeaſe as mild as *the moſt favourable caſes* of inoculated ſmall-pox?

2. Is the cow-pox contagious; or rather, (to be more familiar) is it communicable from one perſon to another by any other means than that of abſolute inoculation?

3. Is not a perſon, who has had the cow-pox, ever after in complete ſecurity againſt the ſmall-pox, however expoſed to them; and, vice verſâ, having had the latter, equally unſuſceptible of the former?

4. Does the inoculation of the genuine cow-pox, in any inſtance, produce maturated puſtules elſewhere than at the place of inſertion?

5. Is it not highly probable, ſupported by every analogy, and now confirmed by ſome experience, that the vaccine inoculation, which excites ſo little diſturbance in the human ſyſtem, is vaſtly leſs liable to derange its healthy economy, or call up conſtitutional affections, than the variolous inoculation, which, it cannot be denied, eſpecially in ſome families, (I might inſtance my own) is frequently violent, ſometimes dangerous, and now and then fatal?

6. What, therefore, has been the general ſubſequent ſtate of health of perſons, who had the cow-pox ſome years ago?

7. Are ſcrophulous, cutaneous, or other diſeaſes, more prevalent among them, than thoſe who have not been ſubjects of the cow-pox?

8. And, *particularly*, are there any complaints obſervable among, and confined to them, of a new and peculiar character; or *any impreſſions whatever*, left on their conſtitutions which can with the leaſt ſhadow of probability be referred to vaccine excitement?

And, laſtly, Is there not the ſtrongeſt probability that the ſwine and the chicken pox derived their origin, at ſome diſtant period, from the animals whoſe names they take; and that hence it is as probable the inoculated cow pox, having ariſen in the

ſame fame way, will hereafter engage as little of our attention as thefe mild difeafes do at prefent?

---

To the EDITORS of the MEDICAL and PHYSICAL JOURNAL.

Gentlemen,

FROM the mafs of evidence already before the medical world, and from what has fallen under my own obfervation, I am entirely difpofed to give credit to the prefent advantages faid to be derived from the new inoculation; and to thofe much greater confequences which promife to refult from it to pofterity. I experienced the greateft fatisfaction on finding a man of Dr. Denman's great and juftly acquired celebrity expreffing himfelf fo difpaffionately on the important fubject of cow-pox. His letter will undoubtedly give a weighty fupport to the intereft of vaccine inoculation; and I cannot help obferving, that I feel pleafed by his remark, "I have thought that fome good might be produced by an attempt to remove prejudices;" for does it not feem to refer to fome fuch means as thofe I have taken liberty to fuggeft? Flattered, indeed, I fhall be, fhould the enclofed queries, and the mode of conveying them to the public, firft through the channel of your truly valuable Journal, and thence by extracts through that of the moft popular prints, at all meet the ideas of Dr. Denman. I have turned the fubject in my mind with as much affiduity and intereft as my prefent avocations will admit of, and nothing has certainly occurred to me, which promifes to be equally ufeful as a preliminary means of fixing public attention on thofe, I earneftly hope, interefting facts. The cow-pox will foon, I truft, be met and treated with the fame indifference that the fwine and chicken-pox now are: if therefore, at the fame time, proper methods were adopted to oppofe the fpread of the fmall-pox when, and wherever, they make their appearance, they would in all probability, in no very long time, be difpoffeffed of their prefent tenure, and be only fpoken of as a difeafe that had been.

I again make it a requeft, that you will give the enclofed (if you think them worth it) as early an infertion as poffible.

I am, GENTLEMEN,
With great refpect,
Your obedient humble fervant,
RICHARD DUNNING.

